# Association between Sleep Deprivation in Caregivers and Risk of Injury among Toddlers: A Propensity Score Analysis

**DOI:** 10.1155/2020/9421712

**Published:** 2020-06-22

**Authors:** I-Tsung Chiu, Ting-Yun Huang, Jiann Ruey Ong, Ju-Chi Ou, Ping-Ling Chen, Hon-Ping Ma

**Affiliations:** ^1^Department of Emergency Medicine, Shuang-Ho Hospital, Taipei Medical University, New Taipei City, Taiwan; ^2^Emergency Medicine, School of Medicine, Taipei Medical University, Taipei, Taiwan; ^3^Graduate Institute of Injury Prevention and Control, College of Public Health, Taipei Medical University, Taipei, Taiwan

## Abstract

**Introduction:**

Unintentional injury remains the leading cause of death in children worldwide. Adequate parental supervision is a crucial strategy for preventing injury. Many factors, such as a large family size, poor socioeconomic status, and the caregiver being a single mother, contribute to unintentional injury in children. In addition, sleep deprivation in caregivers might be associated with injury in children because sleep deprivation causes impaired daytime cognitive function, wake-state instability, and negative moods, thereby impairing caregiver supervision. Therefore, this study determines the association between injury in children and the sleep quality of their primary caregivers.

**Method:**

This is a retrospective case–control study on unintentional injury in children aged 0 to 4 years who visited the emergency department (case group) and an age- and sex-matched control group. Sleep quality in caregivers was assessed using the Chinese version of the Pittsburgh Sleep Quality Index (PSQI). Logistic regression was used to evaluate the association between aspects of the PSQI and injury. A propensity score model was used to generate a quasirandomized design.

**Results:**

This case-control study recruiting 277 injured and 274 noninjured children was conducted in Taiwan. There was no statistically significant difference in child's age and primary caregiver's age between the injured and noninjured groups. The primary outcome, Pittsburgh sleep quality index, was not significantly different between the two groups. The average scores of sleep duration and habitual sleep efficiency in the control group were higher than those in the case group. However, there was no difference between the two groups after adjusting via a propensity score model, including the following potential confounders, child's age, child's sex, number of previous injury, caregiver mental status, caregiver's sex and caregiver's age, and the number of children living together.

**Conclusion:**

Our study was the first to examine the association between injury in children and the sleep quality of their primary caregivers. We observed that no PSQI component significantly affected the risk of injury among children.

## 1. Introduction

Unintentional injury remains the leading cause of death in children, although mortality caused by infectious diseases has decreased as a result of vaccination and the use of antibiotics [1]. However, death is neither the only nor the most common result of injury; according to a survey conducted by the United Nations International Children's Emergency Fund, for every child that dies of an injury, 12 children are admitted to a hospital or disabled and 34 children require medical care or miss school [2]. In addition to causing mortality and disability, injury alone causes an enormous medical burden on society. In the United States, approximately 9.2 million children visit emergency departments (EDs) because of unintentional injury every year [3].

Injury in children can be prevented and controlled; however, the stages of child development should be considered in injury prevention strategies [1]. As children develop, their physical and cognitive abilities change gradually. However, children are often more curious than they are competent in understanding and responding to accidents [4]. Infants and toddlers are more vulnerable to injuries than are children in other age groups.

Relevant studies have revealed that several socioeconomic factors are related to the risk of injury in children, such as maternal age and education level, family income, and family structure (single parent or large family) [5, 6]. These factors increase the risk of injury for various reasons, such as inadequate supervision, inadequate safety equipment, and unsafe living conditions [1, 7]. Among the prevention strategies, supervision is considered crucial for protecting children from harm [1]. Numerous factors affect the quality of supervision. Maternal depression, socioeconomic deprivation, and excessive alcohol consumption may result in inadequate supervision [8]. In addition, sleep deprivation might be a factor related to inadequate supervision that causes an increase in the incidence of injuries.

Sleepiness and fatigue increase the probability of human error caused by a decrease in attentiveness [9, 10]. Poor sleep quality, short sleep duration, and excessive daytime sleepiness are risk factors of work performance, quality of parental supervision, and injuries in children. [9, 11, 12]

Most studies have focused on the characteristics of children with injuries instead of the characteristics of caregivers. However, sleep deprivation impairs daytime cognitive function and mental health, and it leads to inadequate parental supervision. Consequently, sleep deprivation in caregivers might increase the risk of injury in infants and toddlers. There is no related study that investigate the relationship between the sleep quality in caregivers and injury in children. Therefore, this study determined the association between injury in children and the sleep quality of their primary caregivers.

## 2. Methods

### 2.1. Participants

**Figure 1:**
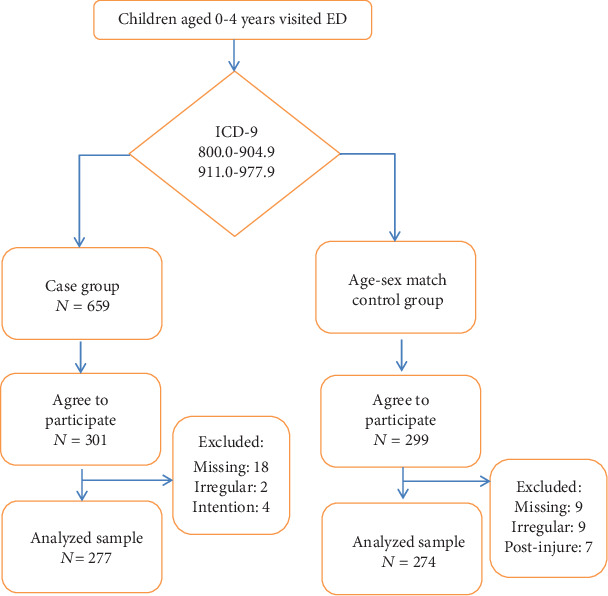
Flow chart.

This was a retrospective case–control study. The study population consisted of the primary caregivers of children aged 0 to 4 years who had visited the ED at Shuang-Ho Hospital between September 1, 2013, and March 31, 2014. According to the primary International Classification of Diseases, Ninth Revision, Clinical Modification (ICD-9-CM) codes, which is the current classification of diseases used in Taiwan, the children were assigned to either the case group or the control group. The children were included in the case group if they were assigned the ICD-9 codes 800.0 to 904.9 or 911.0 to 977.9, which include injuries of the head, spine, chest, and abdomen as well as those pertaining to limb contusions, bone fractures, superficial wounds, burns, crushing, and animal bites. The injury was determined to be unintentional according to a review of medical charts, which were created by the emergency physicians based on the clinical course and type of injury; the participant (primary caregiver of the injured child) was present at the scene of the accident, which was verified using telephonic interviews. The children who were not assigned ICD-9 codes 800.0 to 904.9 or 911.0 to 977.9 belonged to the control group. In addition, the children in the control group were matched with the children in the case group according to sex and age (see Figure 1).

Children with attention deficit hyperactivity disorder, a chronic disease, or developmental delay were excluded from both groups. Children whose caregivers exhibited abnormal sleeping times, which was defined as mainly working at night and sleeping in the daytime, were excluded because results obtained with these children cannot be applied to the general population. In addition, the children in control group who had sustained an injury during the year preceding the study period were excluded.

Telephone interviews were conducted within 1 month after the first visit to the ED. In the case group, 301 of 659 (45.68%) caregivers were contacted and agreed to participate, and 277 (92.03%) completed the survey; in the control group, 299 caregivers were contacted and agreed to participate, and 274 (91.64%) completed the survey. The following variables were documented: caregiver age, caregiver sex, ED injury–related visit before interview (previous injury), caregiver mental status, and number of children living together. Satisfactory caregiver mental status was defined as the absence of the following problems: difficulty of falling asleep or feelings of tenseness, sadness, irritability, or inferiority to others.

### 2.2. Sleep Quality of Caregivers

Sleep quality during the month before the accident was evaluated for primary caregivers using the Chinese version of the Pittsburgh Sleep Quality Index (PSQI) [13], which was translated from the English version [14]. PSQI consists of 19 items to create 7 aspects and to summarize one globe score. The seven aspects are duration of sleep, sleep disturbance, sleep latency, habitual sleep efficiency (HSE), daytime dysfunction, use of sleep medication, and overall sleep quality. The last two aspects are evaluated directly via its corresponding questions. The score of “Duration of sleep” is 0 for sleeping more than 7 hours, 1 for 6-7 hours, 2 for 5-6 hours, and 3 for less than 5 hours. Poor “Sleep disturbance” means you have trouble to sleep frequently, such as fel cold or hot, or pain. Poor “sleep latency” means more time to fall asleep or frequently cannot get to sleep within 30 minutes. Habitual sleep efficiency (HSE) is calculated as percentage of the total number of hours asleep divided by the total number of hours in bed, and it is scored 0 for more than 85%, 1 for 75%-84%, 2 for 65%-74%, and 3 for less than 65%. The score for each aspect ranges from 0 (no problems) to 3 (severe problems), and the global score of the PSQI ranges from 0 to 21. A high score indicates poor sleep quality.

### 2.3. Statistical Methods

Continuous variables were described using means and standard deviations, and the normality of data was evaluated using the Shapiro–Wilk test. The differences between the case and control groups were evaluated using the Student *t*-test for normally distributed data and the Mann–Whitney *U* test for abnormally distributed data. Categorical variables were expressed as frequencies and percentages, and the chi-squared test was used to compare the two groups. A propensity score method was adopted to account for potential confounding factors and thereby generate a quasirandomized design in order to balance two nonequivalent groups on observed covariates [15]. The propensity score model included the following potential confounders: child age, sex, and number of previous injuries; caregiver mental status, sex, and age; and the number of children living together. The associations between sleep aspects and injury in the case and control groups were evaluated using logistic regression analyses. A *P* value of <0.05 indicated statistical significance. Statistical analyses were performed using R software version 3.5.3.

## 3. Results

The control and case groups comprised 274 and 277 children, respectively (Table 1). In the case group, the average age of the 163 (58.8%) boys and 114 (41.2%) girls was 1.92 years. In the control group, the average age of the 150 (54.7%) boys and 124 (45.3%) girls was 2 years. The average ages of the primary caregivers of children in the control and case groups were 35.92 and 37.25 years, respectively, and this age difference was nonsignificant. The numbers and percentages of female caregivers in the control and case groups were 202 (73.7%) and 161 (58.8%), respectively, and the difference in these percentages was statistically significant. The numbers and percentages of caregivers with at least one mental illness in the control and case groups were 43 (18.6%) and 19 (6.8%), respectively, and the difference in these percentages was statistically significant.

The global score (PSQI), which was 4.29 for caregivers in the control group and 4.00 for those in the case group, did not differ significantly between the two groups (*P* = 0.41). Two of the seven aspects, namely, sleep duration and HSE, exhibited significant differences between the groups. The number of caregivers who experienced sleep disturbances and had poor HSE was higher in the control group than in the case group.

**Figure 2:**
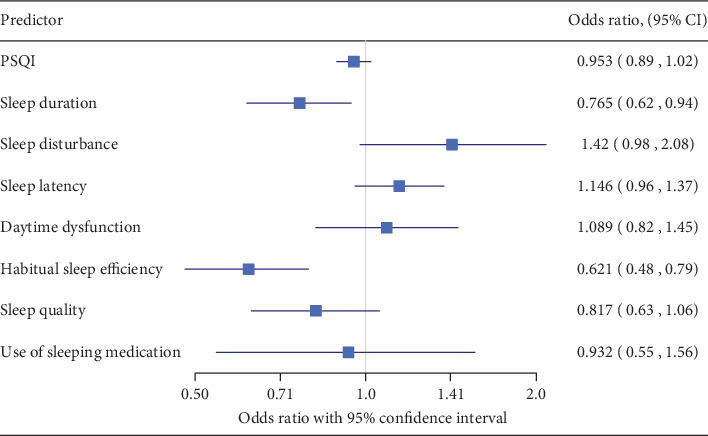
Odds ratios before adjusting according to propensity score.

The crude odds ratios are presented in Figure 2. The risk of injury in children whose primary caregivers experienced sleep disturbances was lower than that in the control group (odds ratio: 0.765; 95% confidence interval: 0.62, 0.94). The risk of injury tended to be lower in children whose caregivers had poor HSE (odds ratio: 0.621; 95% confidence interval: 0.48, 0.79).

**Figure 3:**
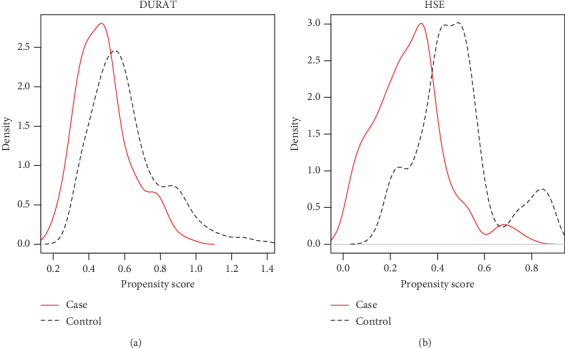
Distribution of propensity score. (a) Duration; (b) habitual sleep efficiency.

**Figure 4:**
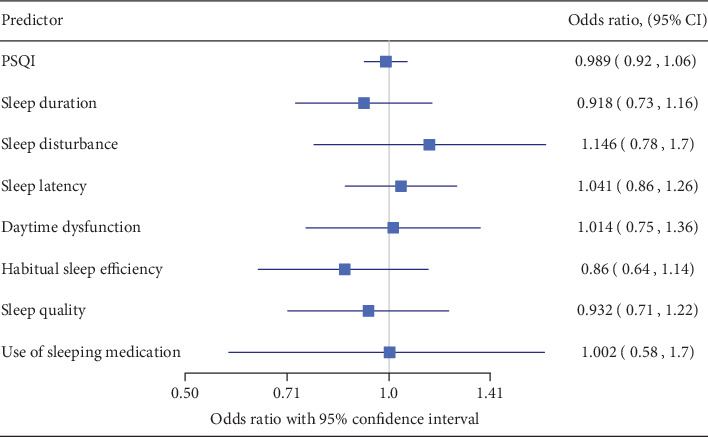
Odds ratios after adjusting according to propensity score.

In Figure 3, the distribution of propensity scores between the case group and control group is shown for sleep duration (Figure 3(a)) and HSE (Figure 3(b)), and the results of the logistic regression models with propensity scores adjustment are shown in Figure 4. After adjusting according to propensity scores, none of the PSQI components differed significantly between the case and control groups. Hence, after adjusting the confounders, the likelihood of being in the case or control group was not affected by the sleep deprivation of the caregiver, which was evaluated using the PSQI global score as well as its components.

## 4. Discussion and Conclusion

Although we did not identify a significant difference in overall sleep quality between the two groups, additional analysis revealed that the caregivers in the control group had a shorter sleep duration and lower HSE than did those in the case group. Parents in the noninjury group may spend time to prevent their children from injuries during night time, but this resulted in shorter sleep duration and poor HSE. However, after adjustment according to propensity scores, none of the PSQI components were significantly associated with injury in children in the case or control group.

Research has proven that sleep deprivation increases the risk of accidents attributable to human error [16]. Numerous questionnaires and physical examinations have been developed to evaluate sleeping conditions. The PSQI is a vital instrument for identifying subjective insufficiency in sleep. However, different sleeping disorders are scored differently in the PSQI. For example, people with excessive daytime sleepiness (EDS) receive higher scores in the daytime dysfunction, sleep disturbance, and subjective sleep quality components than do people with normal sleep quality and those who have difficulty initiating and maintaining sleep [13]. Therefore, analyzing the seven components of the PSQI can provide more information regarding the relationship between the sleep quality of caregivers and unintentional injury in children than can analyze total PSQI scores. However, no significant relationship was observed between injury in children and caregiver PSQI global scores or scores in the PSQI components in our study after adjustment according to propensity scores.

Sleepiness and fatigue increase the probability of human error caused by a decrease in attentiveness [9, 10]. Another study showed that poor sleep quality and short sleep duration impair work performance, including the cognitive and mood-related domains of work performance [9]. A study reported that EDS and sleep quality were two significant independent risk factors for unintentional injury. This relationship also applied to injuries other than those sustained during traffic accidents, which require higher levels of vigilance and judgement [17]. These findings imply that EDS affects the risk of injury through the quality of parental supervision, leading to an increased risk of injury in children. After adjusting background confounders according to the propensity scores, no significant relationship was observed between injury in children and EDS. This research only studied caregivers working at a regular shift, and it is worth to study caregivers working at an irregular shift in the future. In addition, the difference in caregivers' metal health between two groups was found and it may be another important factor to result in an injury in children.

### 4.1. Limitations

This study has some limitations. First, the study is retrospective; hence, recall bias might be present. However, considering the nature of injury, bias was inevitable. Second, all children examined in this study visited the ED. Therefore, caution should be exercised when generalizing the results to children whose caregivers did not seek medical care. In addition, the children visited a single hospital in an urban area; therefore, caution should also be exercised when generalizing the results to populations in suburban or remote areas. Furthermore, because this study was a hospital-based case–control study, selection biases were present, particularly because the participants in the control group consisted of caregivers of children who required medical attention instead of caregivers of healthy children. Approximately half of the caregivers could not be reached or refused to participate in the phone interviews; therefore, the results might have been underestimated.

This is the first study to examine the association between caregivers' sleep quality and injury requiring medical attention in children. However, the mechanism underlying this association remains unknown, and its clarification requires further analysis.

## Figures and Tables

**Table 1:** Comparison of demographic characteristics between the case and control groups (mean ± standard deviation).

Variables	Control group	Case group	*P* value
Sample size	274	277	
Child			
Age (years)	2 ± 1.22	1.92 ± 1.16	0.51
Girls (*n*, %)	150 (54.7%)	163 (58.8%)	0.19
Previous injury	1 (0.3%)	248 (89.5%)	<0.01∗
Number of kids who live together	1.66 ± 0.73	1.64 ± 0.6	0.76
Caregiver			
Age	35.92 ± 7.41	37.25 ± 8.2	0.068
Women (*n*, %)	202 (73.7%)	161 (58.8%)	<0.01∗
Mental illness (*n*, %)	43 (18.6%)	19 (6.8%)	<0.01∗
PSQI	4.29 ± 2.63	4.00 ± 2.30	0.41
Sleep duration	0.64 ± 0.86	0.46 ± 0.74	0.02∗
Sleep disturbance	0.85 ± 0.49	0.92 ± 0.40	0.05
Sleep latency	0.77 ± 0.90	0.88 ± 0.97	0.18
Daytime dysfunction	0.34 ± 0.60	0.37 ± 0.57	0.29
Habitual sleep efficiency	0.50 ± 0.83	0.26 ± 0.62	<0.01∗
Sleep quality	1.16 ± 0.69	1.07 ± 0.60	0.25
Use of sleeping medication	0.05 ± 0.37	0.04 ± 0.30	0.79

^∗^ *P* < 0.05; PSQI: Pittsburgh Sleep Quality Index.

## Data Availability

The data are available from the corresponding author upon request (PL. Chen:plchen@tmu.edu.tw).
